# The complete mitochondrial genome of oceanic puffer (*Lagocephalus lagocephalus*) from South China Sea

**DOI:** 10.1080/23802359.2019.1688098

**Published:** 2019-11-12

**Authors:** Lei Xu, Xuehui Wang, Feiyan Du

**Affiliations:** a South China Sea Fisheries Research Institute, Chinese Academy of Fishery Sciences, Guangzhou, China;; b Guangdong Provincial Key Laboratory of Fishery Ecology and Environment, Chinese Academy of Fishery Sciences, Guangzhou, China

**Keywords:** Mitochondrial genome, *Lagocephalus lagocephalus*, the South China Sea

## Abstract

*Lagocephalus lagocephalus* is one of genus *Lagocephalus* and widely distributed in tropical and temperate oceans. In this study, we described the complete mitochondrial genome of *L. lagocephalus*. The genome is 166,443 bp in length, encoding the standard set of 13 protein-coding genes (PCGs), 22 transfer RNA genes, 2 ribosomal RNA genes, and a non-coding D-loop, with circular organization. The overall base composition of the whole mitochondrial genome was A (27.95%), T (25.07%), G (16.14%), and C (30.83%) with an AT bias of 53.03%. The longest PCG of these species was *ND5*, whereas the shortest *ATP8*.

Puffers of family Tetraodontidae are commercially valuable and highly regarded as food fish. The genus of *Lagocephalus* is characterized by having an elongated and streamlined body, a relatively elongated and blunt snout, a distinct ridge of skin along the ventrolateral part of the body. Most species of *Lagocephalus* are distributed in the temperate and tropical sea. Eight known species or subspecies of the genus *Lagocephalus* are distributed in the South China Sea (Wu et al. 2011). *Lagocephalus lagocephalus* is circumglobal species and occurs in all tropical and temperate oceans and the Mediterranean Sea (Erguden et al. 2017).

Here, we sequenced and annotate mitogenome of *L. lagocephalus* form South China Sea to provide molecular information for genetically understanding of oceanic puffer. The specimens of *L. lagocephalus* were collected from the South China Sea (20°37′N, 115°51′E) in 19 August 2017. Whole genomic DNA was extracted from muscle tissue of one specimen of *L. lagocephalus* using TIANamp Marine Animals DNA Kit (TIANGEN, China). The concentration for use as a PCR template was adjusted to an A_260_ of about 0.05–0.2. The collected specimen and extracted DNA were stored in Guangdong Provincial Key Laboratory of Fishery Ecology and Environment (specimen accession number: DS2017-S2-B6). The complete mitochondrial genomes of *L. lagocephalus* was sequenced using PCR primers designed from highly conserved regions of transfer RNA (tRNA) sequences of related species (Yamanoue et al. 2009) with additional specific primers designed as required from sequences already obtained. Long-PCR amplifications were performed by thermo-cycling using five pairs of primers and PCR amplicons were subjected to build up genomic library and pair-end sequencing by MiSeq. The COI sequence of *L. lagocephalus* was used as reference seeds for iterative assembly by MITObim v.1.8 (Hahn et al. 2013). SeqMan v.7.1.0 was used for the mitogenome assembly and annotation (Swindell and Plasterer 1997). tRNA genes were predicted using online software tRNAScan-SE 1.21 (Lowe and Eddy 1997). All protein-coding genes (PCGs) are aligned independently, then concatenated to be applied for phylogenetic reconstruction with other Scombriformes in MrBayes v 3.12 (Ronquist and Huelsenbeck 2003) using relaxed clock model.

The *L. lagocephalus* mitochondrial genome forms a 16,443 bp closed loop (GenBank accession number MN244304). The overall base composition of the whole mitochondrial genome was A (27.95%), T (25.07%), G (16.14%), and C (30.83%) with an AT bias of 53.03%. This mitochondrial genome represents a typical *Lagocephalus* mitochondrial genome and matches with the *L. wheeleri* genome (Yamanoue et al. 2009), in which it comprises 13 PCGs, 22 tRNA genes and 2 ribosomal RNA genes (12S rRNA and 16S rRNA) and one A + T-rich region which could also be termed as control region. The ATG initiation codons are used in all PCGs except *COX1* (GTG), and the stop codons of all the 13 PCGs were complete. Meanwhile, the longest PCG of these species was *ND5* (1809 bp), whereas the shortest *ATP8* (162 bp). *lrRNA* and *srRNA* genes are 1665 bp and 950 bp in length separately, and the length of D-loop is 817 bp. All the 22 typical tRNAs possess a complete clover-leaf secondary structure, ranging from 64 bp to 73 bp. The Bayesian inference phylogenetic tree showed that *L. lagocephalus* firstly grouped with species of *L. laevigatus* (Figure 1). We have the confidence to construct phylogenetic trees, based on the complete the mitochondrial genomes, but the evolution history of puffer fish still needs future research to be clearly resolved.

**Figure 1.**
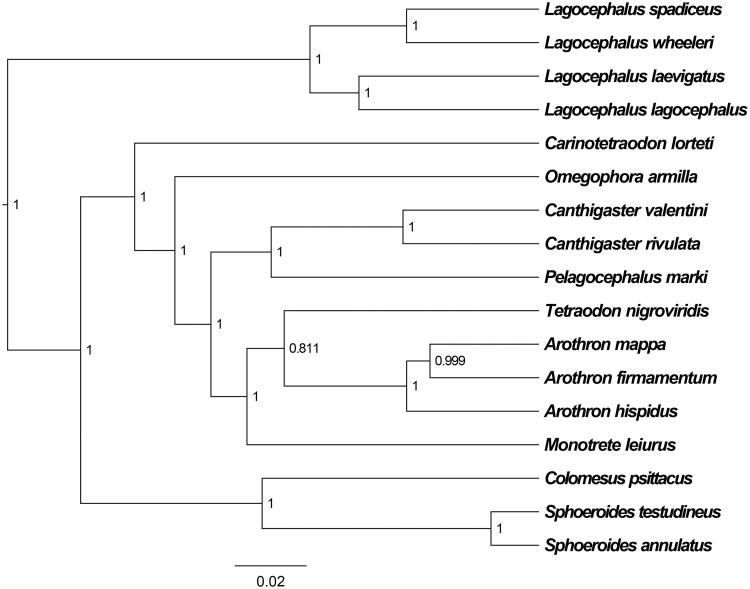
The Bayesian inference phylogenetic tree for Tetradontoidea based on mitochondrial PCGs and rRNAs concatenated dataset. The gene’s accession numbers for tree construction are listed as follows: *Lagocephalus laevigatus* (AP011934), *Lagocephalus wheeleri* (AP009538), *Lagocephalus spadiceus* (KM667972) *Arothron hispidus* (AP011930), *Arothron firmamentum* (AP006742), *Arothron mappa* (AP011931), *Canthigaster valentini* (AP011912), *Tetraodon nigroviridis* (AP006046), *Canthigaster rivulata* (AP006744), *Monotrete leiurus* (KF667490), *Sphoeroides testudineus* (AP011916), *Sphoeroides annulatus* (AP011915), *Carinotetraodon lorteti* (AP011918), *Colomesus psittacus* (AP011910), *Pelagocephalus marki* (AP011938), *Omegophora armilla* (AP011936).

## References

[CIT0001] Erguden D, Gurlek M, Turan C 2017 First occurrence of the oceanic puffer, *Lagocephalus lagocephalus* (Linnaeus, 1758) in Iskenderun Bay, north-eastern Mediterranean, Turkey. J Appl Ichthyol. 33(4):801–803.

[CIT0002] Hahn C, Bachmann L, Chevreux B 2013 Reconstructing mitochondrial genomes directly from genomic next-generation sequencing reads-a baiting and iterative mapping approach. Nucleic Acids Res. 41(13):e129. 23661685 10.1093/nar/gkt371 PMC3711436

[CIT0003] Lowe TM, Eddy SR 1997 tRNAscan-SE: a program for improved detection of transfer RNA genes in genomic sequence. Nucleic Acids Res. 25(5):955–964. 9023104 10.1093/nar/25.5.955 PMC146525

[CIT0004] Ronquist F, Huelsenbeck JP 2003 MrBayes 3: Bayesian phylogenetic inference under mixed models. Bioinformatics. 19(12):1572–1574. 12912839 10.1093/bioinformatics/btg180

[CIT0005] Swindell SR, Plasterer TN 1997 Seqman, contig assembly In: Swindell SR, editor. Sequence data analysis guidebook. Totowa (NJ): Springer; p. 75–89. 9089604

[CIT0006] Wu RX, Liu J, Ning P 2011 A new record species of the head rabbit puffer, *Lagocephalus Lagocephalus* (Linnaeus, 1758) from China seas. Acta Zootaxonom Sin. 36(3):622–626.

[CIT0007] Yamanoue Y, Miya M, Matsuura K, Miyazawa S, Tsukamoto N, Doi H, Takahashi H, Mabuchi K, Nishida M, Sakai H 2009 Explosive speciation of Takifugu: another use of fugu as a model system for evolutionary biology. Mol Biol Evol. 26(3):623–629. 19074759 10.1093/molbev/msn283

